# Importance of hospital cancer registries in Africa

**DOI:** 10.3332/ecancer.2019.948

**Published:** 2019-07-25

**Authors:** Maria Paula Curado

**Affiliations:** A C Camargo Cancer Center, rua Tagua 440 Liberdade, Sao Paulo, CEP 01508-010, Brazil; International Prevention Research Institute, Dardily 69570, France

**Keywords:** hospital cancer registry, cancer, Africa

## Abstract

The establishment of effective population-based cancer registries (PBCRs) in low-resource countries is challenging. There is a lack of knowledge among cancer patients who do not go to treatment centres, there is an absence of mortality information, frequently employed as a complementary back-up in cancer registries and a lack of efficient and accurate population census. Hospital-based cancer registries (HBCRs) have a different, although complementary role: they focus more on clinical information about patients and are sources of cancer information about cancer diagnosis, treatment and survival in African countries. Establishing and sustaining an HBCR in a cancer centre or cancer treatment unit can provide data about the mode of diagnosis, the clinical features of the tumour, treatment and follow-up details. In addition, the HBCR can be a sustainable source to help sustain local cancer-control programmes. The HBCR can also be a source of information for PBCRs.

## Background

Information about the cancer burden on the African continent is scarce, due to limited local resources, the availability of accurate information regarding the number of cancer patients, the basis of the cancer diagnosis and the availability of accurate data about the population. As an indicator of cancer burden, population-based cancer registries (PBCRs) are the main tool to identify incident cases in a geographical area for a defined population, and they can support the development and evaluation of cancer-control programmes [1]. As of 2006, almost 80% of the world’s populations were not covered by PBCR and most of these were in low- and middle-income countries [2]. Data from the African continent are widely lacking. In the most recent volume of Cancer Incidence in Five Continents (volume XI), there were seven PBCRs with data from Africa [3]. Even with initiatives from WHO/International Agency for Research on Cancer (IARC) [4] to improve cancer incidence data by implementing regional training courses and setting regional hubs (https://gicr.iarc.fr/iarc-regional-hubs-for-cancer-registration/) to enhance and strength cancer registration, the instability and continuity of the PBCRs remains challenging.

## Hospital-based cancer registries

Nevertheless, to treat cancer patients, many African countries have dedicated cancer hospitals or cancer units in general hospitals. These hospitals receive patients generally from the urban and rural areas around the treatment area. According to Young [5], hospital-based cancer registries (HBCRs) can be fundamental sources of information in limited resource regions, where population based cancer registries are lacking. They can give an indication of the demand for cancer care services, which is useful for health policy and planning [5]. In addition, cancer hospitals are a part of cancer-control programmes and are an essential component of the health care system [6].

Therefore, in a continent like Africa, it can be worthwhile to establish hospital cancer registries (HCRs) at the cancer centres and units of diagnosis and treatment in order to gather information on cancer diagnosis, treatment and survival. For example, in Brazil, there is a legal requirement that all cancer treatment units have an HBCR: a database on the patient characteristics, such as age, gender, address, educational level, tobacco and alcohol consumption, tumour stage, data of diagnosis, treatment adopted and survival [7]. Knowing that the main sources of data on cancer are the medical records, it is possible to register detailed data such as: admission date, diagnosis date, stage, treatment and outcomes (recurrence, cure and death). To achieve a reliable HBCR, a coordination by the local physician at the cancer centre is needed, as well as training the cancer registrar in case finding, abstracting and registering the cancer cases in an organized data base containing the essential variables related to the patient and the tumour [8].

Thus, it should be possible to create a reliable database from validated medical records in the units of cancer or cancer centres in Africa. In addition, a continuous collaboration with other cancer centres in the country can facilitate the exchange of reliable data, leading to improving cancer care in the area and gathering important survival information. The human resources (cancer registrars) can be trained continuously through online modules, for example, from very well-known sources, such as Surveillance, Epidemiology, and End Results (SEER, https://seer.cancer.gov/) from the USA [9] or on site by periodical cancer registration courses. An online forum can be established with experts in cancer registration. Thus, it is feasible to implement HBCRs in Africa to accelerate information on cancer from African cancer centres aiming to create a network of cancer centres with HBCRs.

**Figure 1.**
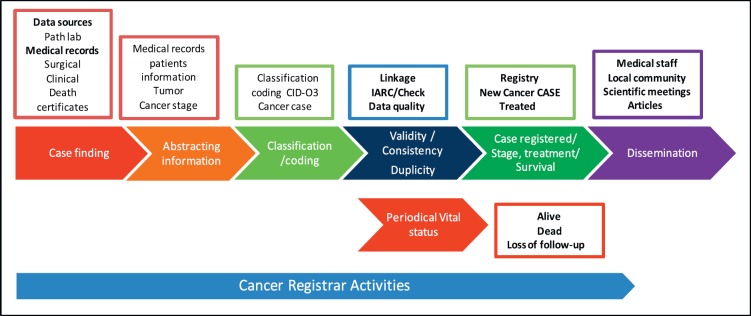
Flow chart to register a cancer case at an HBCR.

Figure 1 shows a description of the standard operation procedures to be followed by the cancer registrar in an HBCR to register a cancer case.

## Conclusion

All too often, cancer presents at an advanced stage in Africa when cure is no longer possible and palliation is the sole alternative. Many groups and organisations are working to improve the outcome of cancer in African patients. This requires information about the cancer stage and mode of diagnosis, the treatment employed and the follow-up information. The use of HBCRs as sources of cancer information can improve knowledge on cancer diagnosis, treatment and survival in African countries. Establishing and sustaining an HBCR in a cancer centre or cancer treatment unit is paramount to produce cancer registration with good data quality. In addition, the HBCR can be a sustainable source to sustain local cancer-control programmes and can be a complementary source of information to PBCRs.

## Conflicts of interest

The author has no conflicts of interest to declare.

## Funding

The author received no funding for this article
